# Clinical outcomes associated with complementary and alternative medicine-related “immunity-boosting” practices in patients with cirrhosis during the COVID-19 pandemic – an observational study

**DOI:** 10.1097/MD.0000000000033365

**Published:** 2023-03-24

**Authors:** Cyriac Abby Philips, Arif Hussain Theruvath, Resmi Raveendran, Rizwan Ahamed, Sasidharan Rajesh, Jinsha K Abduljaleel, Ajit Tharakan, Philip Augustine

**Affiliations:** a Clinical and Translational Hepatology, The Liver Institute, Center of Excellence in GI Sciences, Rajagiri Hospital, Aluva, Kerala, India; b Complementary and Alternative Medicine (Homeopathy), Department of Clinical Research, The Liver Institute, Center of Excellence in GI Sciences, Rajagiri Hospital, Aluva, Kerala; c Complementary and Alternative Medicine (Ayurveda), Department of Clinical Research, The Liver Institute, Center of Excellence in GI Sciences, Rajagiri Hospital, Aluva, Kerala, India; d Gastroenterology and Advanced GI Endoscopy, Center of Excellence in GI Sciences, Rajagiri Hospital, Aluva, Kerala, India; e Interventional Radiology, Center of Excellence in GI Sciences, Rajagiri Hospital, Aluva, Kerala, India.

**Keywords:** ayurveda, AYUSH, cirrhosis, DILI, herb-induced liver injury, homeopathy

## Abstract

During the coronavirus disease 2019 pandemic, Ayurvedic herbal supplements and homeopathic immune boosters (IBs) were promoted as disease-preventive agents. The present study examined the clinical outcomes among patients with chronic liver disease who presented with complications of portal hypertension or liver dysfunction temporally associated with the use of IBs in the absence of other competing causes. This single-center retrospective observational cohort study included patients with chronic liver disease admitted for the evaluation and management of jaundice, ascites, or hepatic encephalopathy temporally associated with the consumption of IBs and followed up for 180 days. Chemical analysis was performed on the retrieved IBs. From April 2020 to May 2021, 1022 patients with cirrhosis were screened, and 178 (19.8%) were found to have consumed complementary and alternative medicines. Nineteen patients with cirrhosis (10.7%), jaundice, ascites, hepatic encephalopathy, or their combination related to IBs use were included. The patients were predominantly male (89.5%). At admission, 14 (73.75%) patients had jaundice, 9 (47.4%) had ascites, 2 (10.5%) presented with acute kidney injury, and 1 (5.3%) had overt encephalopathy. Eight patients (42.1%) died at the end of the follow up period. Hepatic necrosis and portal-based neutrophilic inflammation were the predominant features of liver biopsies. IB analysis revealed detectable levels of (heavy metals) As (40%), Pb (60%), Hg (60%), and various hepatotoxic phytochemicals. Ayurvedic and Homeopathic supplements sold as IBs potentially cause the worsening of preexisting liver disease. Responsible dissemination of scientifically validated, evidence-based medical health information from regulatory bodies and media may help ameliorate this modifiable liver health burden.

## 1. Introduction

Coronavirus disease 2019 (COVID-19), caused by severe acute respiratory syndrome coronavirus 2, was first identified in Wuhan, China, in December 2019, and has since spread worldwide, leading to an ongoing pandemic. On November 24^th^, 2021, the total number of global cases was 258,164,425, with 5,166,192 confirmed deaths.^[1]^ Several action plans, both tested and novel, have been employed worldwide to contain the spread, morbidity, and mortality associated with this challenging new contagion. In addition to enforced and self-imposed lockdowns and “test–trace” isolation methods, face masks, social distancing, and hand hygiene proved valuable for curbing the spread until vaccination the most effective prevention mode against COVID-19 became available.^[2,3]^ Nonetheless, social, visual, and print media were filled with advertisements promoting “immune boosters” (IBs) that claimed to promote “health and wellness” and helped prevent COVID-19 without any validated evidence to support these claims.^[4,5]^ The hype over IBs was so high that some governments, mostly in developing countries, promoted and included complementary and alternative medicine-related supplements such as IBs in their national guidelines for preventing and treating COVID-19. In India, traditional and alternative practices such as Ayurveda and homeopathy play major roles in supplying IBs to the public. Their use is specifically promoted in immunosuppressed patients, such as those with chronic liver disease (CLD).^[6]^ From a scientific standpoint, vaccination is the only measure that can “teach the immune system to boost itself” and limit the disease transmission, severity, and death caused by COVID-19. The present study aimed to characterize and analyze outcomes among patients with known or newly diagnosed CLD who presented with new-onset or worsening portal hypertension or an adverse liver-related event, such as hepatocellular jaundice or hepatic encephalopathy (HE), temporally associated with the use of IBs.

## 2. Methods

### 2.1. Patients

This study examined the clinical outcomes of IB-related adverse events in patients with cirrhosis. We retrospectively included all patients aged > 18 years admitted to the inpatient, high-dependency unit, and intensive care departments from April 2020 to May 2021 for evaluation and management of jaundice, ascites, HE, or combinations temporally associated with the consumption of complementary and alternative medicine (CAM) for COVID-19 prevention with the meticulous exclusion of other competing causes. All patients were followed for 180 days or until death or liver transplantation, whichever occurred first. A thorough diagnostic workup for acute events and CLD was performed for all patients. Jaundice was defined as bilirubin levels > 3.5 mg/dL, and grade ≥ 2 ascites or overt HE was considered clinically significant. Other well-recognized causes of liver injury and acute decompensations were excluded based on laboratory tests for bacterial infections, viral serologies (including a nucleic acid test for severe acute respiratory syndrome coronavirus 2, hepatitis B and hepatitis C viruses, and other acute hepatotropic viruses), and diagnostic imaging. Autoimmune markers, including antinuclear antibody, anti-smooth muscle antibody, anti-liver kidney microsome type 1, and serum IgG levels, were measured in all patients. Liver biopsy was performed in patients who consented to histopathological evaluation and was mandatory in those with suspected CLD during the current presentation. The Roussel Uclaf Causality Assessment Method score and the resulting causality grading were used to diagnose IB-related liver events as follows:1 to 2, unlikely; 3 to 5, possible; 6 to 8, probable; and ≥ 9, highly probable. Patients were excluded due to active alcohol consumption in the preceding 3 months, CAM use for reasons other than COVID-19 prevention, treatment-naive patients newly diagnosed with hepatitis B or C infections, autoimmune hepatitis, non-cirrhotic portal hypertension, extrahepatic or hepatic malignancy, tumoral or benign portal vein thrombosis, critically ill cirrhosis patients, and use of concomitant, known hepatotoxic prescription drugs. The Institutional Review Board of the Center of Excellence in Gastrointestinal Sciences, Rajagiri Hospital, Aluva (Kochi), approved the study protocol. This study was conducted following the Declaration of Helsinki (1975). The Institutional Review Board waived the requirement for informed patient consent because of the retrospective analysis of the existing clinical data.

### 2.2. Statistical analysis

Statistical analyses were performed using the MedCalc Statistical Software (Ostend, Belgium). Continuous variables are expressed as medians with 95% confidence intervals and interquartile ranges, medians with range, or mean and standard deviation, depending on the normality of the data. Categorical variables are summarized as counts and percentages. One-way analysis of variance was used to test for baseline differences between the means of the investigational variables of the groups. *P* values < .05. The probability of patients surviving up to the study endpoints was calculated using the Kaplan–Meier method and graphically represented as the survival time curve. The log-rank test was used to compare the survival curves, and *P* values < .05 were considered significant.

### 2.3. Analysis of retrieved samples

Heavy metal contamination, potential hepatotoxic adulterants, volatile organic compounds, inorganic impurities, insecticides, and pesticides were analyzed in all IB drug samples retrieved from patients using a standard validated methodology. Heavy metal concentrations were determined using an inductively coupled plasma-atomic emission spectrometer (IRIS Intrepid II XSP Duo; Thermo Electron Corp., Munich, Germany) using chemical standards, reagents, and vials according to the United States Environmental Protection Agency standard methods 5021A, 8015, 8021, and 8260. Full-scan qualitative analyses were performed using gas chromatography coupled with tandem mass spectrometry (GC–MS/MS; Thermo Fisher Scientific, Waltham, MA). Pesticide residue analysis was performed using a triple quadrupole GC–MS/MS (GC TRACE 1300 with TSQ EVO 8000 MS).

## 3. Results

### 3.1. Patients

**Figure 1.**
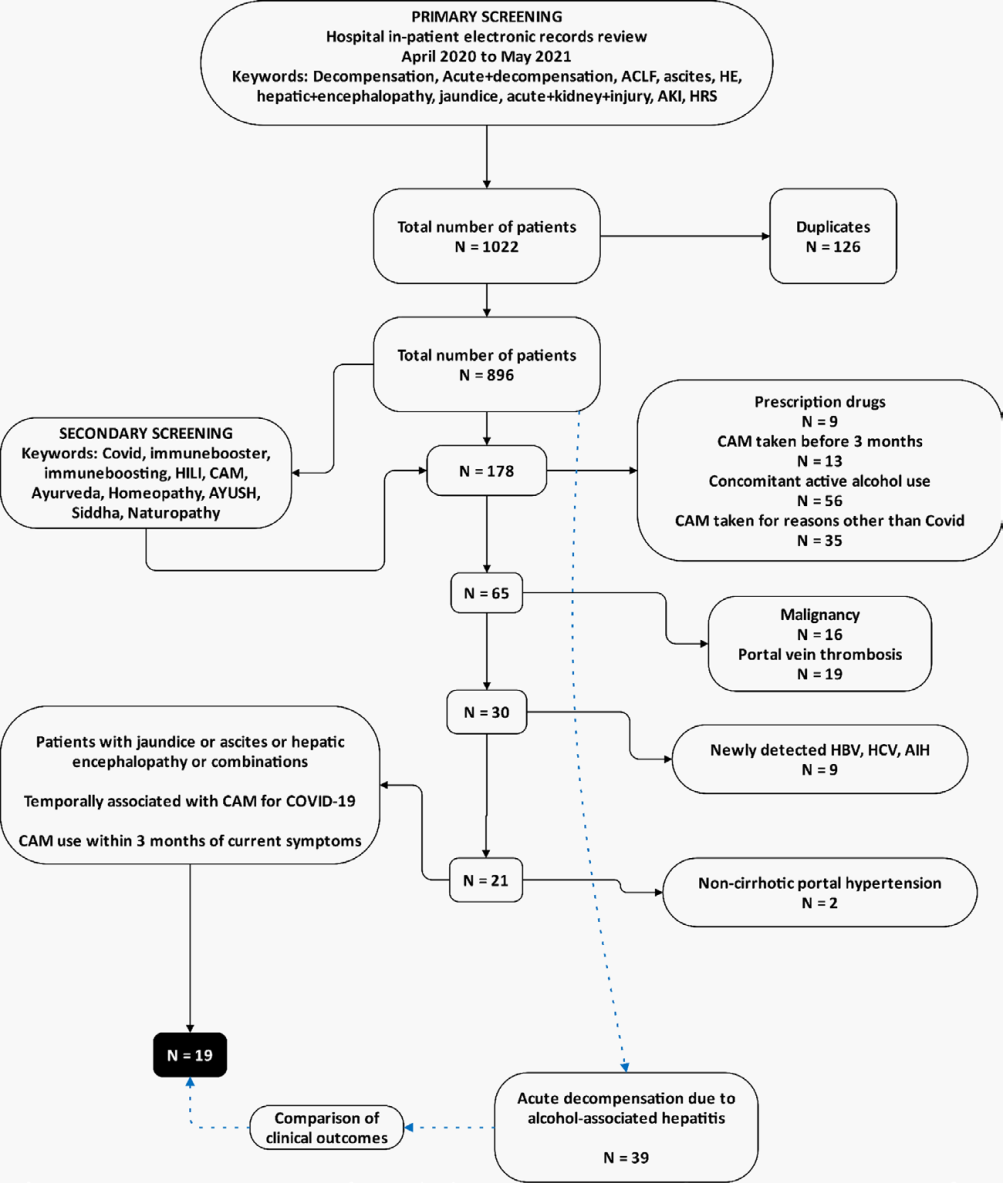
Study flow diagram for patient inclusion, screening, grouping, and final cohort selected for analysis.

Between April 2020 and May 2021, 1022 patients with decompensated cirrhosis were screened for inclusion. Duplicate records (n = 126) were removed, and 896 patients were screened for CAM use. One hundred seventy-eight patients (19.8%) were found to have used CAM before the current admission. Among these, 113 patients were excluded for concomitant hepatotoxic prescription drugs, and CAM use 3 months before the current presentation, significant alcohol consumption, and CAM use for reasons other than COVID-19 prevention. A further 46 patients were excluded due to underlying malignancy, portal vein thrombosis, newly detected and treatment-naive chronic hepatitis B and C viral infections, autoimmune hepatitis, and non-cirrhotic portal hypertension. Nineteen patients with cirrhosis (n = 178, 10.7%) and jaundice, ascites, HE, or a combination of these decompensations, temporally associated with IBs use for COVID-19 prevention within 3 months of symptomatic presentation were included for analysis (Fig. 1). These patients were compared with 39 consecutive patients with decompensation due to biopsy-proven severe alcohol-associated hepatitis (AH) during the same period concerning liver disease severity and clinical outcomes.

### 3.2. Clinical presentation

The study population was predominantly male (n = 17, 89.5%), with a median age of 53 years. The most common etiology of underlying CLD was alcohol consumption (n = 9, 47.4%), followed by nonalcoholic fatty liver disease (n = 7, 36.8%). Sixteen (84.2%) patients had compensated cirrhosis, and 3 (15.8%) had controlled decompensation before admission. Diabetes mellitus was notable in 21% of the study population; 10.5% had hypothyroidism or cardiac disease, and 5.3% were overweight or obese. At admission, 14 patients (73.75%) had jaundice, 9 (47.4%) had ascites, 2 (10.5%) presented with acute kidney injury, and 1 (5.3%) had overt HE at presentation. Cholestatic symptoms were observed in 13 (68.4%) patients at presentation. At admission, 2 patients (10.5%) required renal replacement therapy and mechanical ventilation. The mean ± standard deviation total bilirubin level was 6.1 ± 3.5 mg/dL, and the median Child–Turcotte–Pugh model for end-stage liver disease, chronic liver failure organ failure, and chronic liver failure consortium scores were 11, 21, 8, and 56, respectively. Only 2 patients were antinuclear antibody-positive, with titers of 1:40 and 1:100, respectively, and neither fulfilled the diagnostic criteria for autoimmune hepatitis. None of the patients had serum immunoglobulin G levels > 1.5 above the normal upper limit. The Roussel Uclaf Causality Assessment Method score for the causality of IB use and associated decompensation in patients with cirrhosis was possible (score 3–5) for IB-induced injury in 10.5% and probable (score 6–8) in 89.5% of patients. Baseline investigational parameters are listed in Table 1.

**Table 1:** Baseline characteristics of chronic liver disease patients developing immune boosters related liver injury (N = 19).

	Minimum	Maximum	Mean		Median	SD
Age (yr)	43	73	55.6		53	9.5
Hemoglobin (g/dL)	8.2	14.5	10.8		10.8	1.8
Total counts (x10^3^/μL)	3.9	14	7.4		6.6	3.1
Platelet counts (x 10^6^/μL)	34	346	99.6		83	70.4
Total bilirubin (mg/dL)	1.5	15	6.1		5.1	3.5
Direct bilirubin (mg/dL)	0.3	8.6	2.9		2.6	2.4
Total protein	5.8	9.3	7.01		6.9	0.9
Serum albumin	2.3	3.8	2.8		2.7	0.5
Aspartate aminotransferase (IU/L)	37	487	92.4		67	98.5
Alanine aminotransferase (IU/L)	14	577	74.4		40	123.9
Alkaline phosphatase (IU/L)	70	441	188.5		159	95.3
Blood urea (mg/dL)	14	54	26.3		24	10.5
Serum creatinine (mg/dL)	0.5	2.4	1.04		0.9	0.5
Serum sodium (meq/L)	126	139	132.6		133	3.7
Serum potassium (meq/L)	3.1	4.9	3.9		3.9	0.4
International normalized ratio	0.9	3.9	2.2		2.2	0.7
Child Turcotte Pugh score	6	14	10.1		11	1.8
Model for end-stage liver disease score	8	28	21.6		21	5.5
Chronic liver failure – organ failure score	7	10	8.3		8	1.1
Chronic liver failure – consortium score	37	77	54.8		56	10.8
Gender	Males 17 (89.5%)			Females 2 (10.5%)		
Metabolic disease associations	Diabetes: 4 (21.1%), systemic hypertension: 1 (5.3%), hypothyroidism 2 (10.5%), cardiac disease 2 (10.5%), overweight/obese 1 (5.3%)					
Etiology of chronic liver disease	Alcohol – 9 (47.4%), nonalcoholic fatty liver – 7 (36.8%), hepatitis B virus infection – 2 (10.5%), primary biliary cholangitis – 1 (5.3%)					
Previously compensated chronic liver disease	16 (84.2%)					
Clinical features at presentation/admission	Jaundice 14 (73.7%), ascites 9 (47.4%), hepatic encephalopathy 1 (5.3%), acute kidney injury 2 (10.5%), mechanical ventilation 2 (10.5%), renal replacement therapy 2 (10.5%)					
Roussel uclaf causality assessment model score (total score - % of patients)	5–10.5% 6–10.5% 7–36.8% 8–42.1%					

### 3.3. Follow up and clinical outcomes

During follow up, 11 (57.9%) patients with IB-related decompensation of cirrhosis developed recurrence or new-onset ascites, 9 (47.4%) developed acute kidney injury, 7 (36.8%) had acute variceal bleeding, 8 (42.1%) had overt HE, and 10 (52.6%) were admitted for the intensive care management of critical illness, including infections. The most common critical illnesses were sepsis and overt HE in 4 (n = 10, 40%). During the initial admission, 2 patients (10.5%) underwent renal replacement therapy. Decompensations were unstable in 11 patients (57.9%), among whom 8 (42.1%) died due to portal hypertension and liver failure complications during the 180-day follow up period.

### 3.4. IBs and liver histopathology of IB-related liver injury

The most common IBs consumed by patients (n = 6/19, 31.6%) were marketed Ayurvedic multiherbal products (minimum 2, maximum 6, and median 2 products per patient), which commonly contain herbal ingredients, such as *Tinospora cordifolia* (giloy, heart-leaved moonseed), *Withania somnifera* (ashwagandha, Indian winter cherry), *Ocimum tenuiflorum* (tulsi, holy basil), *Azadirachta indica* (neem, Indian lilac), curcumin (from turmeric, *Curcuma longa*), and some minerals. Other common IB practices included the consumption of decoctions of *Phyllanthus emblica* (amla, Indian gooseberry; n = 6, 31.6%), *Trigonella foenum-graecum* [methi, fenugreek, (n = 4, 21.1%)], guava leaf extracts (n = 1, 5.3%), extracts from the leaves of neem and *Justicia adhatoda* [vasaka or Malabar nut, n = 3, 15.8%)], and the homeopathic remedy *Arsenicum album* 30C (n = 2, 10.5%). The median duration of IB consumption was 38 days, and the median time from IB initiation to symptom onset was 45 days. The details of the IBs taken by the patients are shown in Table S1, Supplemental Digital Content, http://links.lww.com/MD/I706.

**Figure 2.**
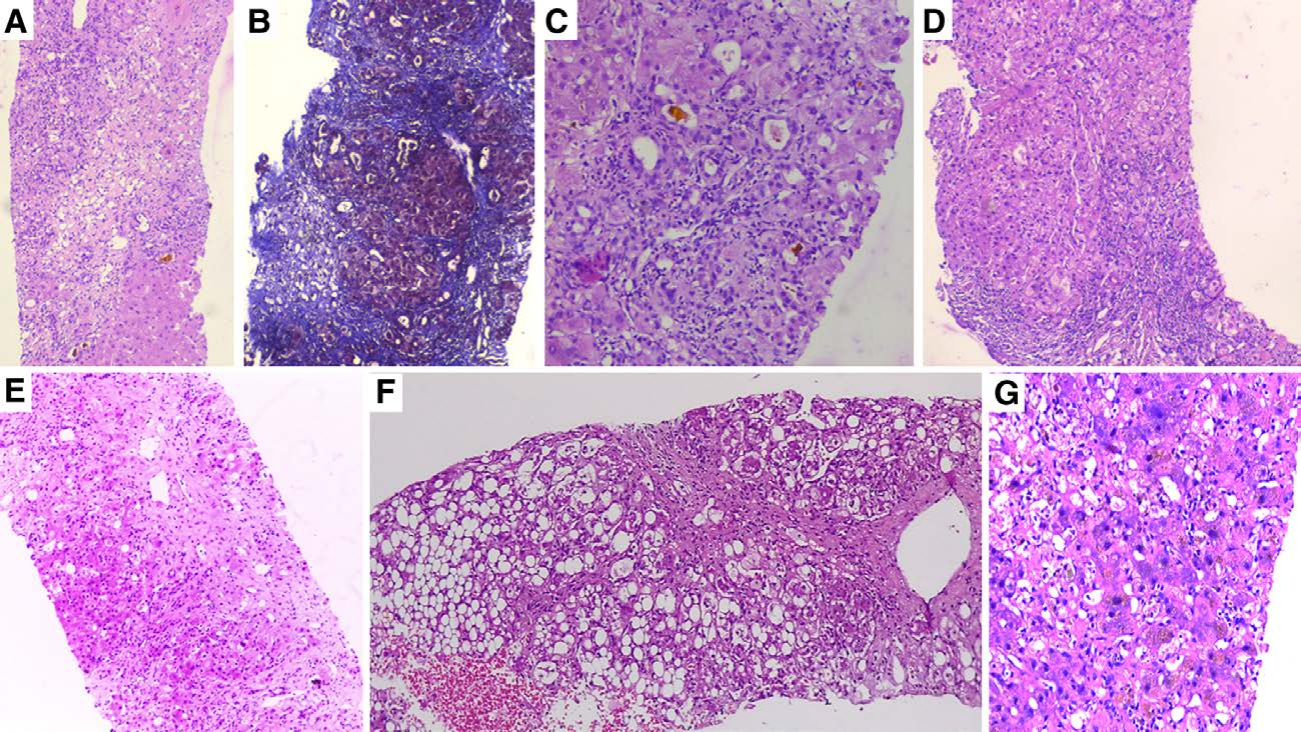
Liver histopathology of chronic liver disease patients with ayurvedic herbal and homeopathic remedy-based liver injury. (A) – zone 3 necrosis bridging with portal area in patient consuming tinospora cordifolia (giloy) and Curcuma longa (turmeric) extract (hematoxylin and eosin, 40x), (B) – cirrhotic nodules with and surrounded by focal areas of necrosis in a patient consuming homeopathic arsenicum album 30C (Masson-trichrome stain, 40x), (C) – cholangiolar cholestasis with surrounding mixed inflammation in a patient consuming multiherbal immune-booster (hematoxylin and eosin, 200x), (D) – bridging fibrosis with severe portal inflammation and interface hepatitis in a cirrhosis patient consuming Indian gooseberry decoction (hematoxylin and eosin, 40x), (E) – multiacinar necrosis with loss of hepatocytes and underlying fibrosis in a patient consuming giloy and withania somnifera (Ashwagandha) containing multiherbal supplement (hematoxylin and eosin, 40x), (F) – severe steatosis and focal necrosis in a patient with cirrhosis consuming Indian gooseberry and guava leaf extract decoction (hematoxylin and eosin, 100x), and (G) – severe hepatocellular cholestasis in a patient with chronic liver disease consuming Ashwagandha extracts (hematoxylin and eosin, 40x).

Seven patients (36.8%) consented to transjugular liver biopsy. Necrosis was notable in all patient samples, and the most common pattern was multiacinar necrosis (n = 3, 42.9%), followed by focal or spotty necrosis (n = 2, 28.6%). Neutrophilic inflammation was predominant (n = 5, 71.4%), and the portal area was the most common site of inflammation (n = 4, 57.1%). Interface hepatitis and cholestasis were observed in 4 (57.1%) patients, canalicular cholestasis was seen in 50%, and canalicular and hepatocellular cholestasis was noted in the other 50%. Hepatocyte ballooning was seen in 6 (85.7%) patients, whereas steatosis was seen in 5 (51.1%) patients. Moderate eosinophilic inflammation was notable in 5 (71.45). Mild portal and perisinusoidal fibrosis were seen in 1 patient, grade 3 fibrosis was seen in 2 patients, and cirrhosis was seen in 4 patients (57.1%). Both patients who developed homeopathic *Arsenicum album* 30C-related liver injury had bridging or confluent hepatic necrosis with the lymphocyte-predominant portal and lobular inflammation compared with neutrophilic inflammation noted in those who took herbal supplements. Liver histopathology findings are shown in Table S1, Supplemental Digital Content, http://links.lww.com/MD/I706, and representative images of IB-related liver injury are shown in Figure 2.

### 3.5. Comparison of clinical outcomes with alcohol-associated acute decompensation

We compared liver disease severity at admission, clinical presentation, follow up clinical outcomes, and survival after IB-related decompensation (n = 19) in consecutive patients with biopsy-proven severe alcohol-associated hepatitis-related decompensation (AH-AD, n = 39). The AH-AD group comprised all (100%) males, compared with 89.5% (n = 17) males in the IB group (*P* = .04). The proportions of patients with ascites, acute kidney injury, and overt HE was similar between the AH and AD, and IB groups at admission, whereas there were differences in jaundice (100% vs 73.7%, respectively; *P* < .001), Child–Turcotte–Pugh (11.4 ± 1.6 vs 10.2 ± 1.9, respectively; *P* = .01), and model for end-stage liver disease scores (26.3 ± 5.1 vs 21.6 ± 5.5, respectively; *P* = .002), which were significantly higher in AH-AD patients. Nonetheless, at day 180 of follow up, new-onset or recurrence of ascites, overt HE, acute variceal bleeding, acute kidney injury (Table S2, Supplemental Digital Content, http://links.lww.com/MD/I707), and survival (66.7% vs 57.9%, *P* = .71, Figure S1, Supplemental Digital Content, http://links.lww.com/MD/I708) were not significantly different between the AH and AD and IB groups.

### 3.6. Analysis of retrieved samples

**Figure 3.**
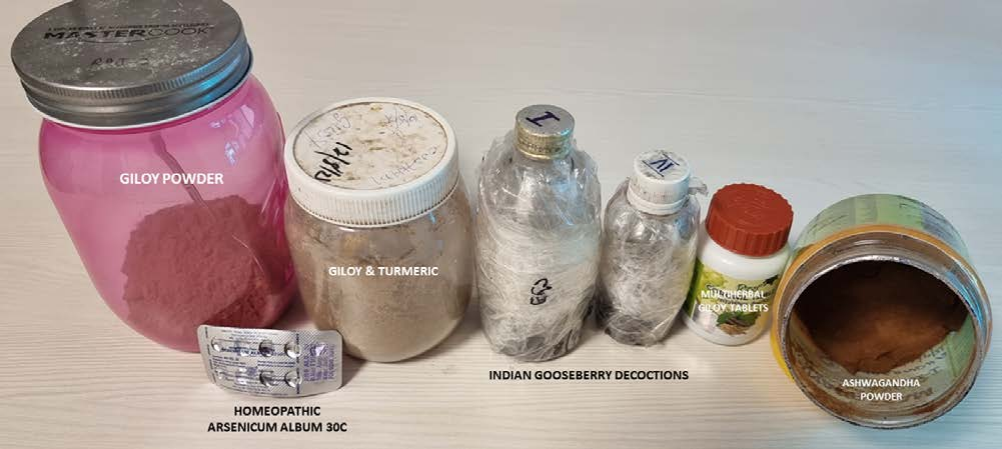
Retrieved ayurvedic herbals and homeopathic remedies from patients with chronic liver disease developing complementary and alternative medicine-related portal hypertension events and liver dysfunction.

Ten samples of IBs, including locally made ashwagandha powder, giloy juice, Indian gooseberry extracts, pure giloy tablets, multiherbal immune-boosting powder, other multiherbal tablets, and the homeopathic remedy, *Arsenicum album* 30C, were retrieved from our study patients (Fig. 3). Samples were analyzed for potential hepatotoxic prescription drugs, known hepatotoxic adulterants, pesticides, and insecticides, which were not present in any of the samples. Detectable levels of arsenic (40%), lead (60%), and mercury (60%) were found in the samples analyzed. A host of other plant-derived compounds, industrial solvents, chemicals, and anticoagulants was identified using GC–MS/MS. These include glycosides, terpenoids, phytosteroids, and sterols, such as sitosterol, lupeol, trilinolein, hydroxy menthol, methoxyphenol, butyl alcohol, and coumaran derivatives. A detailed list of quantified heavy metals and other compounds is provided in Table 2.

**Table 2:** A detailed list of quantified heavy metals and other compounds in Ayurvedic and Homeopathic immune-booster supplements retrieved from cirrhosis patients with liver injury (N = 10).

Product	Arsenic (mg/kg)	Lead (mg/kg)	Mercury (mg/kg)	Other compounds identified on GC-MSMS scan
Ashwagandha powder		6.55	0.32	Sitosterol Lupeol Stigmasterol Campesterol Quassin d- Sesamin Trilinolein 4’-o-methyl glabridin Retrofractamide- A Coronarin E Dihydroxanthin Ambrial Nerolidyl acetate Acorenol Nerolidol Lavandulyl acetate Terpinylacetate Geraniol
Giloy tablets	0.81	5.03	0.38	2-methoxy-4-vinyl phenol Decanoic acid Asarone aR-turmerone Myristic acid Lauric anhydride Palmitic acid methyl ester Estafiatin Columbin Campesterol Stigmasterol Amyrin Sitostenone
Giloy juice				Phenol, 2-. methoxy-5-(2-propenyl)- 2,4-Di-tert-butyl phenol Dihydroxanthin Linolenin,1-mono- 6-epi-shyobunol Nootkaton-11,12-epoxide Estafiatin Estra-1,3,5 (10)-trien-17a’-oL Ingol 12-acetate 29, 25-Dihydroxy cholecalciferol Dihydroagathic acid Methyl 11-octadecenoate Propanoic acid Columbin Palmitin,1,2-di- 3-Hydroxy spirost-8-en-11-1
Indian gooseberry extract 1				n-carpylic acid Estafiatin Phytol Methyl stearate Phthalic acid Columbin Fenretinide Squalene a-Tocopherol c-sitosterol Lupeol Decanoic acid
Indian gooseberry extract 2				Ascaridole epoxide 2,4-Di-tert-butyl phenol Erucic acid Tetra acetyl-d-xylonic nitrile Ascorbic acid Palmitin, 1, 2-di- trans-13-octadecanoic acid cis-13-octadecanoic acid c1-Sitosterol Olean-12-en-3-one
Multiherbal powder	1.55	1.91	0.29	Limonen-6-ol, pivalate 2, 4-Di-tert-butyl phenol Palmitic acid,methyl ester Palmitin, 1, 2-di- Benzoic acid,methyl ester Vanillin lactoside cis-methyl isoeugenol 2, 4-Di-tert-butyl phenol Asarone c1-Asarone Isocalamendiol Tatarinoid B Palmitic acid, methyl ester Isopropyl palmitate Linoleic acid, methyl ester Oleic acid, methyl ester Phytol Methyl stearate Isopropyl linoleate Glycerol 1-palmitate Squalene Vitamin E
Multiherbal tablets–1		0.26	0.44	p-methan-3-one Menthol, trans-1, 3, cis-1, 4- Menthol (n~)- Carvone Menthol acetate, iso- Quinoline, 1, 2-dihydro-2, 2, 4-trimethyl- 2, 4-Di-tert-butyl phenol t-Butylhydroquinone Palmitic acid Olein, 2-mono- c1-Tocopherol Campesterol Stigmasterol c1-Sitosterol
Multiherbal tablets–2		1.08	0.1	Asarone c1-Sitosterol Piperidine trans-1, 2-Diphenyl cyclobutane Amyrin Palmitin Lup-20 (29)-en-3-ol, acetate Normethadol Squalene d-Sesamin Lupenone
Multiherbal tablets–3	0.61	1.63	0.54	Coumaran Vanillin Lactosidea Curcumene aR- Turmerone 6. Ascorbic acid Dehydrocostuslactone Piperidine Flavone Shogaol Squalene Lupeol c1-Sitostenone
Arsenicum album 30C	0.18			Melezitose

## 4. Discussion

**Figure 4.**
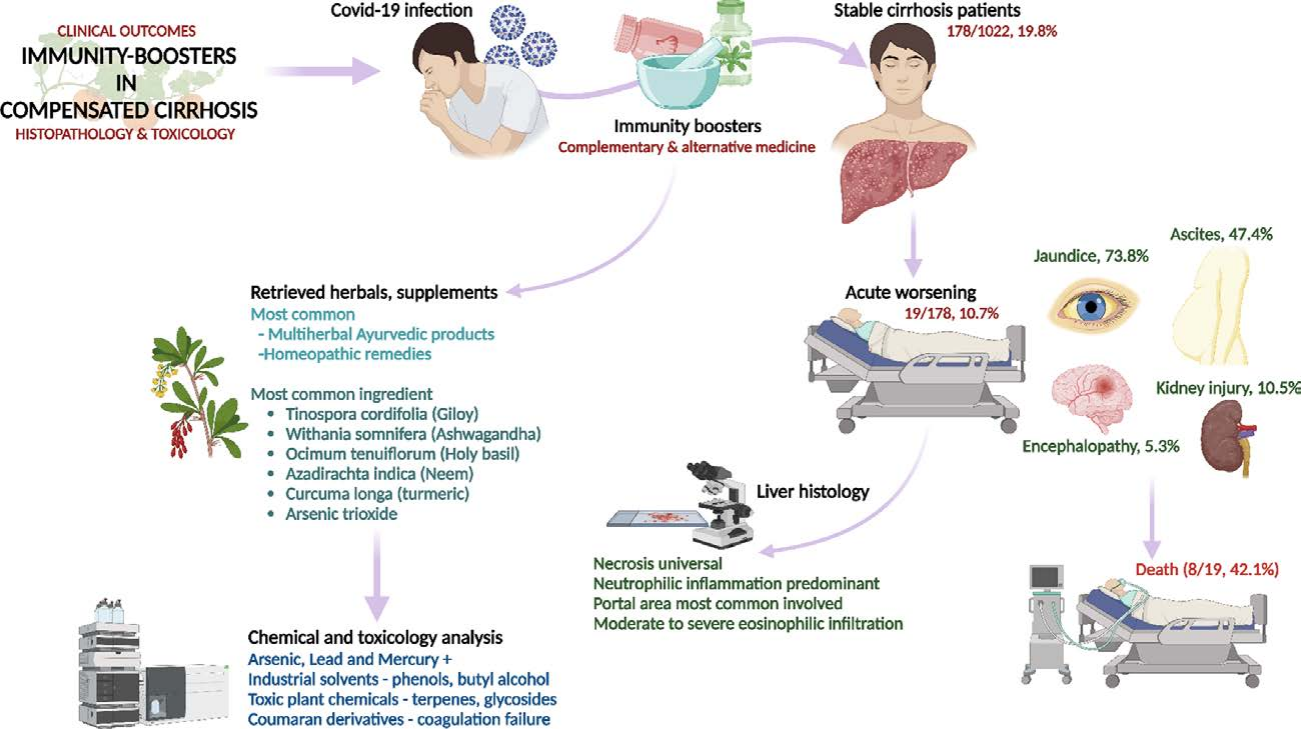
Infographics summary of the study.

The term IB is vague and generalized and is used to label and market products and practices that are “claimed to” improve general immunity and thereby prevent infection. However, there are limited data in animal models for products advertised as IBs and a lack of established clinical efficacy and safety studies in humans.^[7]^ The present study describes the clinical features and outcomes of alternative medicine-related IB practices in COVID-19 patients with CLD. These practices were predominantly Ayurveda-based and included consuming traditional herbal supplements, decoctions, and proprietary and marketed Ayurvedic drugs. All patients had a stable CLD at baseline, and causality assessment tools revealed that decompensation in the form of portal hypertension events or liver failure was temporally associated with IB consumption in the absence of other competing causes. We examined the liver histopathology of patients with herbal and homeopathic supplement-related liver injury and found predominantly neutrophil-type inflammation associated with various types of necrosis. The IB-associated liver injury led to death in 42% of the patients with CLD in our cohort. Exhaustive chemical and toxicological analyses of the retrieved IBs showed detectable levels of heavy metals and a host of other organic, inorganic, and volatile compounds with the potential for liver injury. Compared with patients with severe decompensation due to AH, survival outcomes were similar between the groups at 6 months, demonstrating the high morbidity and mortality associated with CAM-related practices among patients with CLD (Fig. 4, summary infographics)

The Asia–Pacific Association of Study of Liver Research Consortium found that important identifiable causes of the acute worsening of CLD in Asia–Pacific countries were attributed to drugs, predominantly CAM. Encephalopathy, bilirubin, blood urea, lactate, and international normalized ratio could predict mortality in their cohort’s drug-induced acute worsening of CLD.^[8]^ Philips et al^[9]^ showed that HE, hypoalbuminemia, and hepatic necrosis were significantly associated with mortality in patients with severe liver injury related to Ayurvedic herbal supplements containing heavy metals and hepatotoxic volatile organic compounds. Similarly, a single-center study examining the outcomes of CAM use among 1666 patients with cirrhosis revealed that 68% used CAM at some point and 35.7% presented with CAM drug-induced liver injury (DILI)-related decompensation. The most common use of CAM is in unlabeled polyherbal Ayurvedic formulations. Portal-based neutrophilic-predominant mixed inflammation, hepatocyte ballooning, autoimmune-like features, and severe cholestasis were observed on liver biopsy, and 53% of patients died, with a median survival of 194 days.^[10]^ In the present study, IB Ayurvedic formulations were associated with decompensation in patients with underlying CLD, and liver histology in biopsied patients revealed neutrophilic inflammation-predominant hepatic necrosis. This important finding can lead to poor prognosis in the absence of liver transplantation.

Some herbal ingredients consumed by our patients are known to cause liver injury. In studies by Björnsson et al^[11]^ and Weber et al^[12]^, {Ashwagandha-induced liver injury was reported in several patients. Björnsson et al found that liver injury due to Ashwagandha was predominant with multiherbal formulations, typically cholestatic or mixed with severe jaundice and pruritus, but was self-limiting with liver function normalizing within 1 to 5 months. Weber et al described 11 patients who reported liver damage after consuming Ashwagandha extracts (9 with pruritus, 6 with jaundice, and 3 with choluria), with 6 patients requiring hospitalization due to severe hepatitis and 2 developing clinical features of acute liver failure based on self-reported user reviews on commercial websites. The authors also reported more than 107 user-reported reviews on pruritus that developed after ingestion of Ashwagandha extract. A clinical study conducted in India showed that consuming Ayurvedic herbal formulations containing Ashwagandha (330–440 mg per day for 6 weeks) worsened liver function.^[13]^ In the present study, patients consumed multiherbal Ayurvedic formulations predominantly containing Ashwagandha, and cholestatic symptoms were notable in > 60% of patients at presentation. In contrast, cholestatic features were notable on liver biopsy in > 50% of the patients.

Lombardi et al^[14]^ described 7 cases of acute hepatitis due to turmeric ingestion in Tuscany, Italy. Hepatotoxicity was associated with turmeric formulations with high bioavailability and high dosage of curcumin or curcuminoids, and the causal relationship was supported by positive de-challenge in most cases. In 23 cases identified via a systematic review of the Italian Phytovigilance Database, the authors also found that most patients were concomitantly exposed to at least 1 other medication, and 16 patients experienced a positive de-challenge. More cases of turmeric-induced severe hepatocellular injury with rechallenge were reported by Luber et al^[15]^, and a case of turmeric-induced liver injury with autoimmune features was recently reported by Lee et al^[16]^ In our patient cohort, the concomitant use of turmeric or turmeric supplements was evident in most patients who consumed multiple supplements. This would have contributed to idiosyncratic and synergistic liver toxicity in patients with underlying CLD.

The majority of multiherbal supplements consumed by our patient cohort were giloy-based formulations. Recent studies from the Indian subcontinent have demonstrated a strong risk associated with the use of giloy (*Tinospora cordifolia*) and severe liver injury, as well as serological or histological features of autoimmune liver injury. It is difficult to identify a specific ingredient or herbal extract that induces DILI because of the complexity of herbal ingredients and the presence of multiple phytochemicals in the supplements used by most patients. Nonetheless, using multiherbal supplements in the absence of other competing causes is associated with a high risk of liver injury.^[17]^

While the literature is full of foundational evidence on the hepatoprotective role of Indian gooseberry extracts, these findings have not been confirmed in well-designed clinical studies. Using highly concentrated decoction formulations of Indian gooseberry, in the presence or absence of concomitantly used multiherbal products, could lead to idiosyncratic liver injury. Various preclinical and foundational studies on guava leaf extracts have demonstrated their beneficial role as “hepatoprotective” agents, although no supporting translational clinical trials have demonstrated similar findings.^[18]^ In contrast, toxicity studies of the effects of guava leaf extract in mice showed its potential to cause steatosis and hepatocellular injury, as well as dose-dependent inflammation in the liver.^[19]^ A recent study conducted in India on herbal supplements and traditional medicine-induced ACLF demonstrated the presence of hepatocellular necrosis, neutrophilic portal inflammation, and predominantly intracanalicular cholestasis in a patient who consumed guava leaf extract and aloe vera for diabetes.^[10]^

Several studies have shown that monoterpenes, such as limonene, sesquiterpenes, and phytochemical components identified in guava leaf extracts, promote liver toxicity due to the formation of secondary reactive metabolites, increased levels of reactive oxygen species, and impaired antioxidant defense.^[20]^ Preclinical and clinical studies have shown that a multitude of phytochemicals such as anthraquinones, pyrrolizidine, piperidine alkaloids, furan derivatives, furanoid terpenes, diterpenes, triterpenes, quinolines, catechins, glycosides, glucosides, flavonoids, alkylbenzenes, turmerones, and lactones, either alone or in combination, have the potential to cause significant liver damage. Drug-drug and herb–herb interactions can lead to the generation of hepatotoxic secondary metabolites that promote idiosyncratic liver toxicity in those consuming multiherbal products. In the present study, GC–MS/MS analysis of retrieved herbal supplement samples identified a multitude of phytochemicals with the potential to cause liver injury, worsen liver function, and promote liver failure.^[21,22]^

The Asia–Pacific Association for the Study of Liver found that drugs, predominantly CAM followed by antituberculosis drugs, were important identifiable causes of ACLF in Asia–Pacific countries. The authors also found that high mortality was notable in individuals with preexisting liver disease compared to those without underlying liver disease in the United States DILI Network study. It is pertinent to note that classical markers of liver injury, such as elevated levels of aspartate transaminase and alanine transaminase, are unreliable markers of DILI in patients with preexisting liver disease. In such patients, new-onset or worsening jaundice, coagulopathy, and portal hypertension events are more appropriate and indicative of hepatic dysfunction.^[8]^ In our patient cohort, liver enzyme levels were near normal in some patients, and worsening liver synthetic function or portal hypertension events due to CAM IB use in the absence of other competing causes were considered acute events, leading to poor clinical outcomes. In CAM-induced DILI, causality assessment is particularly challenging when patients are exposed to multicomponent products without labelings, such as prescriptions commonly seen in traditional medicines, including Ayurvedic and homeopathic drugs. Poor regulation, manufacturing practices, and safety and efficacy of herbal supplements, such as IBs used in cirrhosis, increase the risk of developing liver injury that leads to new-onset or worsened decompensation.^[23]^

Although patients with AH had greater severity of liver disease and worse clinical profiles at baseline than those who developed IB-associated CLD decompensation, there was no significant difference in the 6-month survival between these patients. This could be due to a lack of standardized, recommended, or known treatment regimens for patients with CAM-related liver injury since, in most cases, the injurious agent was a multiherbal preparation. Identifying and treating actual hepatotoxic components are challenging, leading to the option of best supportive care or liver transplant, contrary to those with severe alcohol-associated liver disease for whom standard recommendations for treatment to improve clinical outcomes exist.^[24–26]^ The high mortality associated with the use of Ayurvedic herbal supplements in patients with underlying liver disease is well-known and documented in a large series and was also notable in our group of patients.^[27]^ We had aimed to identify statistically relevant associations between the CAMs consumed and specifics on liver histology but were unable to do so due to multiple herbal products and multiherbal formulation use among patients and the small sample size.

Complementary and alternative medicines, such as Ayurvedic herbal supplements and homeopathic remedies sold as IBs, potentially induce idiosyncratic liver injury in patients with preexisting liver disease. Using such untested advertised products can lead to the worsening of CLD in the form of liver failure or portal hypertension events, which are associated with a high risk of mortality compared to those with severe AH-related liver decompensation in the absence of timely liver transplantation. Severe mixed portal inflammation and varying levels of hepatic necrosis are common findings on liver histopathology in IB-related liver injury. Health regulatory authorities and print and visual media must ensure the dissemination of responsible and factual scientific evidence-based information on herbal and homeopathic “immune boosters” and health supplements to the public, specifically to the at-risk patient population. This would help ameliorate the modifiable liver health burden within the communities to avoid an epidemic of misinformation-based liver injury during the COVID-19 pandemic.

## Author contributions

**Conceptualization:** Cyriac Abby Philips.

**Data curation:** Cyriac Abby Philips, Arif Hussain Theruvath, Resmi Raveendran.

**Formal analysis:** Cyriac Abby Philips, Arif Hussain Theruvath.

**Methodology:** Cyriac Abby Philips, Arif Hussain Theruvath, Resmi Raveendran.

**Supervision:** Rizwan Ahamed, Sasidharan Rajesh, Jinsha K Abduljaleel, Ajit Tharakan, Philip Augustine.

**Validation:** Rizwan Ahamed, Sasidharan Rajesh, Jinsha K Abduljaleel.

**Writing – original draft:** Cyriac Abby Philips.

**Writing – review & editing:** Cyriac Abby Philips, Rizwan Ahamed, Sasidharan Rajesh, Jinsha K Abduljaleel, Ajit Tharakan, Philip Augustine.

## Supplementary Material






