# Supplementary material for: Clinical outcomes associated with complementary and alternative medicine-related “immunity-boosting” practices in patients with cirrhosis during the COVID-19 pandemic – an observational study
Source: Medicine (Baltimore). 2023 Mar 24;102(12):e33365. doi: 10.1097/MD.0000000000033365 (PMC10035553; doi:10.1097/MD.0000000000033365)
Supplement: Supplementary file 1 [file medi-102-e33365-s001.pdf]

Supplementary Table 1: Details of Ayurvedic and Homeopathic immune-booster supplements consumed by chronic liver disease patients and histopathology findings of those developing liver injury

Immune-booster consumed	Number of patients	Liver histopathology (N = 7)	
**Turmeric, Ashwagandha**	1	***Feature***	***Proportion of patients***
**Turmeric, Giloy juice**	1	Ballooning	85.7%
**Arsenicum album 30C**	2	Cholestasis	57.1%
**Indian Gooseberry decoction**	3	Canalicular and hepatocellular	50%
**Indian Gooseberry, Guava leaf extract**	1	Necrosis	100%
**Indian Gooseberry, Fenugreek decoction**	1	Focal or spotty type	28.6%
**Fenugreek, Turmeric decoction**	1	Bridging type	14.3%
**Multiherbal products (powders, tablets)**	6	Confluent type	14.3%
**Malabar nut, neem, turmeric decoction**	1	Multiacinar type	42.8%
**Ashwagandha, Turmeric and multiherbal**	1	Steatosis	71.4%
**Indian gooseberry, Malabar nut extracts**	1	Eosinophils	100%
**Total**	19	Moderate severity	71.4%
		Inflammation type	Neutrophilic 71.4%

		Inflammation pattern	Portal region 57.1%
		Interface hepatitis	57.1%
		Fibrosis	F2 – 14.3%<br>F3 – 28.6%<br>F4 – 57.1%
