# Supplementary material for: Clinical outcomes associated with complementary and alternative medicine-related “immunity-boosting” practices in patients with cirrhosis during the COVID-19 pandemic – an observational study
Source: Medicine (Baltimore). 2023 Mar 24;102(12):e33365. doi: 10.1097/MD.0000000000033365 (PMC10035553; doi:10.1097/MD.0000000000033365)
Supplement: Supplementary file 2 [file medi-102-e33365-s002.pdf]

Supplementary Table 2: Comparison of clinical events between immune-booster-related decompensation and alcohol-associated acute decompensation at baseline and on follow-up at 180 days

Variable	Immune-booster group (N=19)	Alcohol group (N=39)	P value
**Baseline**			
**Age (in years)**	55.6±9.4	52.6±9.5	0.26
**Gender: Males**	89.5%	100%	0.04
**Child Turcotte Pugh score**	10.2±1.9	11.4±1.6	0.01
**Model for end stage liver disease score**	21.6±5.5	26.3±5.1	0.002
**Ascites**	47.4%	51.3%	0.78
**Acute kidney injury**	10.5%	20.5%	0.34
**Hepatic encephalopathy**	5.3%	41.02%	0.005
**Jaundice**	73.7%	100%	<0.001
**Follow up (180 days)**			
**Ascites**	57.9%	41.02%	0.23
**Acute variceal bleed**	36.8%	33.3%	0.79
**Acute kidney injury**	52.6%	30.8%	0.11
**Hepatic encephalopathy**	42.1%	38.5%	0.80
