# Supplementary material for: Clinical outcomes associated with complementary and alternative medicine-related “immunity-boosting” practices in patients with cirrhosis during the COVID-19 pandemic – an observational study
Source: Medicine (Baltimore). 2023 Mar 24;102(12):e33365. doi: 10.1097/MD.0000000000033365 (PMC10035553; doi:10.1097/MD.0000000000033365)

Supplementary Figure 1: Kaplan-Meier survival analysis and proportion of patients surviving at the end of 180 days follow up between immune-booster related and alcohol-associated decompensation of cirrhosis.

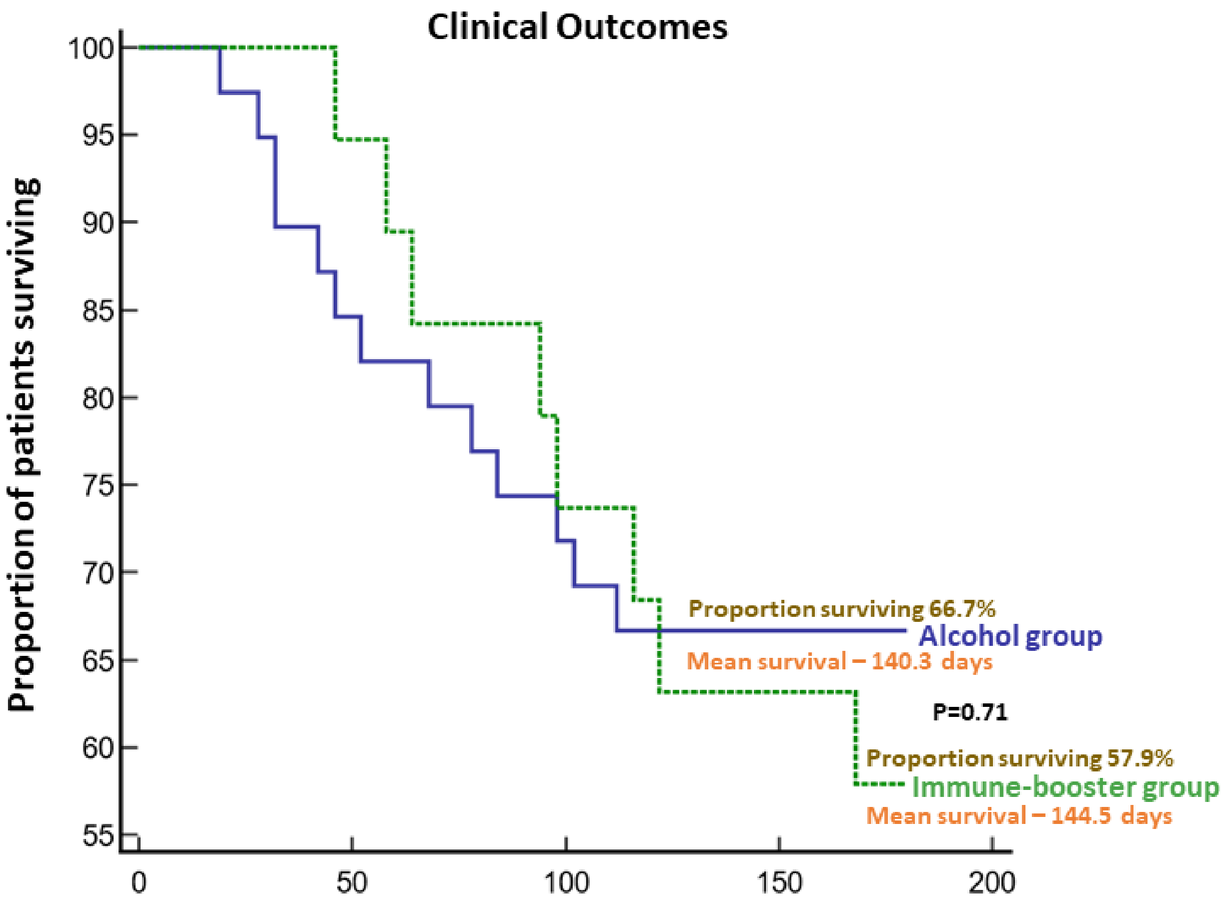

Supplement: Supplementary file 3 [file medi-102-e33365-s003.pdf]
